# Pregnancy outcomes in women with prosthetic heart valves: A single-center study in China

**DOI:** 10.1097/MD.0000000000042622

**Published:** 2025-06-06

**Authors:** Dong Yang, HaoFeng Zhang, Jiaqi Zeng, Xiaojun Liang, Lianmei Luo, Yu Song, Baiyu Tian, Yanna Li, Jie Han, Jun Zhang

**Affiliations:** a Obstetrics and Gynecology, Beijing Anzhen Hospital, Capital Medical University, Beijing, China; b Center for Valvular Heart Disease, Beijing Anzhen Hospital, Capital Medical University, Beijing, China.

**Keywords:** anticoagulation, fetal outcome, maternal outcome, pregnancy, prosthetic heart valve

## Abstract

Accurate information on pregnancy outcomes in women with prosthetic heart valves (PHVs) is essential for preconception counseling and prenatal care. This study aims to determine the maternal and fetal outcomes in women with PHVs. A total of 138 pregnant women with PHVs admitted into a tertiary center between November 2007 and February 2020 were included in the study, and the data were analyzed retrospectively. Patients were divided into the mechanical heart valves (MHVs) group, 118 patients with MHVs, and the tissue heart valves (THVs) group, 20 patients with THVs. The 2 groups were compared. There was 1 maternal death in the MHV group patients (0.8%). There was no difference between the 2 groups regarding maternal mortality (*P* = 1.000), valve thrombosis (*P* = .376), and hemorrhagic events (*P* = .692). Only 66.9% of patients in the MHV group had a live birth compared to the live birth rate of 100% in the THV (*P* *=* .002). Mechanical valve thrombosis (MVT) occurred in 3.2% of patients who used warfarin only; no MVT occurred in patients using a regimen called “sequential therapy”, which utilizes low-molecular-weight heparin (LMWH) during the 1st trimester and warfarin during the 2nd and 3rd trimesters. MVT occurred in 33.3% of patients using LMWH throughout gestation (*P* < .001). The difference was statistically significant. Compared to patients using other regimens, the patients using the regimen of “warfarin only ” was correlated with the highest rate of miscarriage (38.1%, 3.4%, and 16.7%, *P* < .001). Women with MHVs have a lower rate of live birth. The anticoagulation regimen of “sequential therapy” was superior to other regimens in terms of the weighted effects of regimens on maternal MVTs and fetal loss.

## 1. Introduction

Along with the advancement of cardiac surgical techniques, the improvements in the prosthetic heart valve (PHV) design, and the development of anticoagulant agents, the overall prognosis of patients with a PHV has been remarkably improved, including female patients at their reproductive age.^[1]^ The hypercoagulability and the marked hemodynamic changes during pregnancy put high risks on women with a PHV,^[2]^ and fetal development could be compromised by a cardiac complication or the need for cardiac medication.

Given the need for anticoagulation, pregnant women with a mechanical heart valve (MHV) might face more challenges. Doctors have to balance adequate anticoagulation to prevent mechanical valve thrombosis (MVT) against the risks of teratogenicity, fetotoxicity, and bleeding. In China, anticoagulation regimens are mainly categorized as “warfarin only,” “low-molecular-weight heparin (LMWH) throughout gestation,” and “sequential therapy,” which is LMWH during the 1st trimester and warfarin during the 2nd and 3rd trimesters.” Warfarin will be bridged with LMWH 1 week before planned delivery, and LMWH is recommended until 12 hours or 24 hours before delivery.^[3,4]^ But there remains a paucity of data in regard to the impact of different anticoagulation regimens on pregnancy outcomes in Chinese women with a MHV. This study hypothesized that there were significant differences in maternal and infant outcomes during pregnancy among women using different anticoagulation regimens. We speculate that pregnant women using sequential therapy have better maternal and infant outcomes than other anticoagulant regiments, characterized by lower rates of MVT, lower rates of miscarriage, and higher rates of live births. We also hypothesized that patients with mechanical valves had worse pregnancy outcomes than patients with tissue valves, with higher rates of miscarriage and lower rates of live births. Therefore, this study aimed to assess the pregnancy outcomes of women with a PHV, to analyze the risk factors of adverse pregnancy outcomes, and to identify the appropriate anticoagulant regimen for pregnant women with heart valve prostheses.

## 2. Materials and methods

### 2.1. Study population

**Figure 1.**
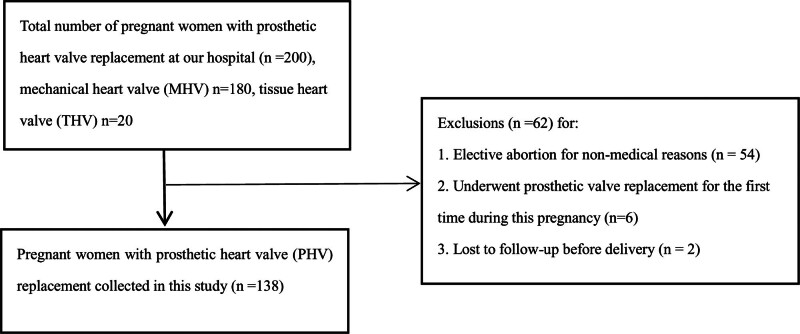
Study population and process of patient enrollment. MHV = mechanical heart valve, PHV = prosthetic heart valve, THV = tissue heart valve.

This retrospective cohort study included pregnant women with heart valve prostheses who were admitted to Beijing Anzhen Hospital between November 2007 and February 2020. Data were extracted from the hospital’s electronic medical record system, and diagnoses were based on the International Classification of Diseases coding. Two hundred cases of pregnancies were identified (Fig. 1), where the patients had heart valve prostheses, and their medical history recorded the valve location and the type of prosthesis. As this article focuses on cardiovascular and pregnancy outcomes, 62 patients were excluded from the analysis because they either selected abortion in the 1st trimester or underwent prosthetic valve replacement for the 1st time during this pregnancy. At the same time, a small number were lost to follow-up during pregnancy. When anticoagulation regimens were analyzed, 2 patients with mechanical prosthetic valves were further excluded because their anticoagulant regimen was not described detailed enough in medical records.

### 2.2. Clinical data

This study has been registered in the Chinese Clinical Trials Registry (chictr.org.cn; registration number: ChiCTR2200056121). Data were collected from detailed medical records of enrolled patients retrospectively. Baseline clinical characteristics included demographic information, reproductive history, surgical history, location and type of prosthetic valve, prior medical use, anticoagulant regimen during pregnancy and perinatal period, and New York heart function classification (New York Heart Association [NYHA]). Anticoagulation regimens during pregnancy were categorized as “warfarin only,” “sequential therapy, with LMWH during the 1st trimester and warfarin during the 2nd and 3rd trimesters,” and “LMWH throughout gestation.”

Warfarin only: Patients in this group were treated with warfarin throughout the entire pregnancy. The dosage of warfarin was adjusted according to the patient’s international normalized ratio (INR) levels, and monitoring was conducted regularly.

Sequential therapy: In this regimen, patients received LMWH during the 1st trimester (weeks 1–12^+6^) to minimize the risk of teratogenicity, as LMWH is considered safer in early pregnancy. From the 2nd trimester (weeks 13–27^+6^) onwards, patients were switched to warfarin, which is more effective in preventing MVT. In the 3rd trimester (weeks 28–40), patients continued with warfarin treatment, with regular INR monitoring to maintain therapeutic levels. Before delivery, LMWH was used as a bridge therapy 1 week before planned delivery to manage anticoagulation around the time of labor.

LMWH throughout gestation: Patients in this group received LMWH throughout the entire pregnancy (from week 1 to 40). LMWH was adjusted based on anti-Xa levels, and no warfarin was used in this group. LMWH was discontinued 12 to 24 hours before delivery to reduce the risk of bleeding complications during labor and delivery.

In this study, adverse maternal outcomes were defined as maternal death or severe morbidity. Severe morbidity included conditions such as valve thrombosis or valve dysfunction requiring medical treatment or cardiac surgery, cerebrovascular accidents (including strokes), infective endocarditis, and hemorrhagic events necessitating blood product transfusion or surgical management. Hemorrhagic events included postpartum hemorrhage, defined as blood loss >500 mL after vaginal delivery or more than 1000 mL after cesarean delivery within the 1st 24 hours postpartum, as well as intra-abdominal bleeding and placental abruption, which is the complete or partial separation of a normally implanted placenta before delivery. Maternal death was defined as death occurring during pregnancy or up to 6 weeks postpartum, consistent with established obstetric definitions.

Fetal outcomes were categorized into several categories: termination of pregnancy before 28 weeks gestation, miscarriage (spontaneous fetal loss before 28 weeks), stillbirth (the delivery of a fetus showing no signs of life, indicated by the absence of breathing, heartbeats, pulsation of the umbilical cord, or definite movements of voluntary muscles at or after 20 weeks gestation), and neonatal death (death of a live-born infant within the 1st 28 days of life). Preterm birth was defined as delivery before 37 weeks gestation, including both spontaneous and iatrogenic preterm births. A live birth was defined as a pregnancy resulting in a live neonate. Apgar score < 7 at 5 minutes after birth was also used as an indicator of neonatal health. Small for gestational age was defined as a birthweight below the 10th customized centile for gestational age. Additionally, other fetal complications were assessed, including fetal malformations and intracranial hemorrhages.

### 2.3. Data analysis

We compared pregnancy outcomes between women with MHVs and women with tissue heart valves (THVs). Secondarily, we assessed the differences in pregnancy outcomes among subgroups based on anticoagulant regimens used during pregnancy and analyzed the factors associated with poor pregnancy outcomes.

The statistical analysis was performed with IBM SPSS Statistics 20.0 (SPSS Inc, Chicago).

Continuous data were described as mean ± SD or as median with 1st and 3rd quartiles range. Kolmogorov–Smirnov tests were used to evaluate data normality. Student *t* test and Mann–Whitney test were used, as appropriate, to study differences between 2 groups, and Kruskal–Wallis H(k) was used to analyze the differences among 3 groups, as appropriate. Categorical data were presented as frequencies and percentages, and the chi-square test or Fisher exact tests were used, as appropriate, to reveal the differences between independent subgroups. Univariable logistic regression analyses were used to assess the clinical parameters and the anticoagulant regimen with poor pregnancy outcomes, followed by multivariable logistic regression analysis, including factors significant in univariate analysis. Results were presented as odds ratios with 95% confidence intervals. To address potential confounding factors, we conducted multivariable logistic regression analysis, which included relevant clinical variables and treatment regimens identified in the univariate analysis. This approach allowed us to adjust for potential confounders and evaluate the independent effects of anticoagulation regimens on pregnancy outcomes. By controlling for these factors, we aimed to improve the reliability of our findings and reduce the bias that might arise from the influence of extraneous variables. For all statistical analyses, a *P*-value < .05 in the context of a 2-sided test was considered significant. Cohen’s d for continuous variables: 0 to 0.20: Negligible; 0.20 to 0.50: Small; 0.50 to 0.80: Medium; 0.80: Large.

### 2.4. Ethics committee approval

Because of the retrospective design of the study, which is based on the data collected for routine clinical administration, the informed consent of the patients was not required. All procedures performed in this study conformed to the 1964 Helsinki Declaration and its later amendments or comparable ethical standards. This study was approved by the Ethics committee of Beijing Anzhen Hospital (2021166X).

## 3. Results

One hundred thirty-eight pregnant women with heart valve prostheses were included in the study. There were 118 patients with MHVs and 20 patients with THVs. The median maternal age was 30 (28–34) years old, with 30 patients (21.7%) being 35 years or older. The median time from prosthetic valve implantation to pregnancy was 6 (3–9) years. One hundred two patients (73.9%) were primiparous, and 36 (26.1%) were multiparous. Ninety-five (68.8%) patients received antenatal care at our hospital since the 1st trimester, while the rest of included patients (n = 43, 31.2%) were transferred to our hospital during pregnancy. Rheumatic valvular disease (n = 65, 47.1%) was the most common cause of valve replacement. Single valve replacement was involved in 116 cases, and mitral position (n = 95, 68.8%) was dominant in this study. Prior to pregnancy, warfarin was used as the anticoagulant agent in 117 patients with MHVs, and LMWH was already given to 1 patient. Fifty-six (40.6%) patients were in functional NYHA class I, while 82 (59.4%) were in NYHA class II. NYHA classification was comparable in patients with MHVs and patients with THVs. Only 9 (31.2%) patients were receiving pharmacological therapies before pregnancy, including β-blocker (n = 7, 5.1%), angiotensin-converting enzyme (ACE) inhibitor (n = 1, 0.7%), and diuretic (n = 1, 0.7%). The baseline characteristics of the patients with heart valve prostheses were shown in Table 1.

**Table 1:** Baseline characteristics of pregnant women with prosthetic valves.

Characteristic	Prosthetic valve (n = 138), n (%)	Mechanical valve (n = 118), n (%)	Tissue valve (n = 20), n(%)	*P*	Effect size (Cohen’s d)
Age, Median (Q1–Q3; yrs)	30 (28–34)	30 (28–34)	28.5 (27–32)	.13	0.02
Age ≥ 35 yrs	30 (21.7)	28 (23.7)	2 (10)	.24	0.08
Parity				.22	0.09
Nulliparous	102 (73.9)	85 (72.0)	17 (85.0)		
Parous	36 (26.1)	33 (28.0)	3 (15.0)		
BMI (kg/m^2^)				.95	0.02
<18.5	16 (11.6)	16 (13.6)	0 (0)		
18.5–24.9	105 (76.1)	89 (75.4)	16 (80)		
≥25	17 (12.3)	13 (11.0)	4 (20)		
Reason for valve replacement				.08	0.21
Congenital heart disease	40 (29.0)	30 (25.4)	10 (50.0)		
Rheumatic heart disease	65 (47.1)	59 (50.0)	6 (30)		
Endocarditis	11 (8.0)	8 (6.8)	3 (15)		
Aortopathy	8 (5.8)	8 (6.8)	0 (0)		
Unknown	14 (10.1)	11 (8.0)	1 (5)		
Location of prosthetic valve				.03	0.71
Mitral	73 (52.9)	64 (54.2)	9 (45.0)		
Aortic	41 (29.7)	33 (28.0)	8 (40.0)		
Tricuspid	2 (1.4)	0 (0)	2 (10)		
Mitral + Aortic	21 (15.2)	20 (16.9)	1 (0)		
Mitral + Aortic + tricuspid	1 (0.7)	1 (0.8)	0 (0)		
NYHA class, n (%)				.16	0.02
I	56 (40.6)	45 (38.1)	11 (55.0)		
II	82 (59.4)	73 (61.9)	9 (45.0)		
Previous medication					
β-Blocker	7 (5.1)	7 (5.9)	0 (0)	.59	0.02
ACE inhibitor	1 (0.7)	1 (0.8)	0 (0)	1.00	0.01
Diuretic	1 (0.7)	1 (0.8)	0 (0)	1.00	0.01

ACE = angiotensin-converting enzyme, BMI = body mass index, NYHA = New York Heart Association.

### 3.1. Obstetric management

All patients in this study received at least 1 multidisciplinary consultation during pregnancy. Overall, 103 (74.6%) patients reached the 3rd trimester of pregnancy (> 28 weeks 0 days gestation). Among these patients, 99 (71.7%) achieved live births, including 79 (66.9% of MHVs) patients with MHVs and 20 (100% of THVs) patients with THVs (*P* < .01). All the 103 patients who reached the 3rd trimester had a perinatal management plan. Eighty-seven (88.3%) patients had a care plan for delivery in their medical records, and 69 (83.1% of MHVs reaching the 3rd trimester) patients with MHVs had an anticoagulation treatment plan during their perinatal period.

Among the 103 patients, 9 of them (8.7%) had a vaginal delivery, and 94 patients (91.3%) underwent a cesarean section (CS) to deliver the baby. In terms of the incidence of CS delivery, it is statistically higher in patients with MHVs than that in patients with THVs (Table 2). Eleven urgent CSs were performed among patients with MHVs (Table 2). Regional anesthesia was used in 85 (90.4%) patients, including epidural anesthesia in 6 (7.1% of regional anesthesia) patients and spinal anesthesia in 79 (92.9% of regional anesthesia) patients. Nine (9.6%) patients with MHVs underwent general anesthesia, including 7 (77.8% of general anesthesia) for unplanned CS, 1 (11.1% of general anesthesia) for cardiac surgery combined lower-segment CS, and 1 (11.1% of general anesthesia) just for a mechanical valve.

**Table 2:** Maternal outcomes and fetal outcomes in women with prosthetic valve.

Characteristic	Prosthetic valve (n = 138), n (%)	Mechanical valve (n = 118), n (%)	Tissue valve (n = 20), n(%)	*P*	Effect size (Cohen’s d)
Maternal outcome					
Maternal death	1 (0.7)	1 (0.8)	0 (0)	1.00	0.03
Heart failure	11 (8.0)	9 (7.6)	2 (10.0)	1.00	0.03
Thrombosed valve	10 (7.2)	10 (8.5)	0 (0)	.38	0.10
Endocarditis	2 (1.4)	2 (1.7)	0 (0)	1.00	0.04
Atrial fibrillation	13 (9.4)	13 (11.0)	0 (0)	.25	0.12
Maternal hospital admission	30 (21.7)	29 (24.6)	1 (5.0)	.08	0.16
Maternal hospital admission for cardiac reason	19 (12.3)	18 (13.6)	1 (5.0)	.48	0.08
Hemorrhagic events	13 (9.4)	12 (10.2)	1 (5.0)	.69	0.05
Postpartum hemorrhage	5 (3.6)	4 (3.4)	1 (5.0)		
Intra-abdominal bleed	1 (0.7)	1 (0.8)	0 (0)		
Placenta bleeding	7 (5.1)	7 (5.9)	0 (0)		
** **Delivery mode					
Cesarean section	94 (91.3)	80 (96.4)	14 (70.0)	<.01	0.85
Urgent	11 (10.7)	11 (13.8)	0 (0)		
Planned	83 (81.6)	69 (86.2)	14 (70.0)		
Vaginal delivery	9 (8.7)	3 (3.6)	6 (30.0)		
Induced onset	3 (2.9)	2 (2.4)	1 (5.0)		
Spontaneous onset	6 (5.8)	1 (1.2)	5 (25.0)		
** **Indication of cesarean section				.43	0.10
CS for Cardiac reason	62 (66.0)	53 (66.2)	9 (64.3)		
CS for obstetric reason	30 (31.9)	26 (32.5)	4 (28.6)		
CS for social reason	2 (2.1)	1 (1.2)	1 (7.1)		
** **Anesthesia for CS				.35	0.11
Regional	85 (90.4)	71 (88.8)	14 (100)		
General	9 (9.6)	9 (11.2)	0 (0)		
Fetal outcome					
Fetal loss	39 (28.3)	39 (33.1)	0 (0)	<.01	0.82
Miscarriage < 28 wk	29 (21.0)	29 (24.6)	0 (0)	.03	0.61
Fetal mortality ≥ 28 wk	3 (2.1)	3 (2.5)	0 (0)	1.00	0.04
Therapeutic abortion: maternal condition	4 (2.9)	4 (3.4)	0 (0)	.91	0.03
Therapeutic abortion and termination of pregnancy: fetal condition	3 (2.1)	3 (2.5)	0 (0)	1.00	0.04
** **Live birth	99 (71.7)	79 (66.9)	20 (100)	.002	0.81
Preterm birth<37 wk	14 (14.1)	13 (16.5)	1 (5.0)	.34	0.10
Median pregnancy duration, wk (Q1–Q3)	37 (37–38)	37 (37–38)	38 (37–38.8)	.04	0.62
Median birth weight, g (Q1–Q3)	2970 (2720–3210)	2960 (2720–3170)	3215 (2830–3535)	.03	0.75
SGA	3 (3.0)	3 (3.8)	0 (0)	1.00	0.04
Apgar < 7 at 5 min	1 (1.0)	1 (1.3)	0 (0)	1.00	0.03
Neonatal death	2 (2.0)	2 (2.5)	0 (0)	1.00	0.04

CS = cesarean section, SGA = small for gestational age.

### 3.2. Anticoagulant therapy

No patient with THVs received anticoagulant therapy. For patients with MHVs, 2 of them who were transferred to our hospital did not provide enough information about their anticoagulant therapy. The other 116 patients with MHVs received anticoagulant therapies, including “warfarin only ” (n = 63, 54.3%), “sequential therapy with LMWH during 1st trimester and warfarin during the 2nd and 3rd trimesters” (n = 29, 25.0%), and “LMWH throughout gestation” (n = 24, 20.7%). Of the 53 patients who used LMWH in the 1st trimester, 1 patient (1.9%) had already been using LMWH before this time of pregnancy; and 10 (18.9%) patients changed to LMWH from other therapies before 8 weeks of gestation; the rest of the patients did not have the information available about the timing LMWH therapy in their 1st trimester. For patients using warfarin, the median dose of warfarin was 3 mg (3–4 mg) daily. About 85.9% (n = 79) of the patients took warfarin at a dose of <5 mg daily, and 6.5% (n = 6) of the patients took warfarin at a dose of more than 5 mg daily. Seven patients (7.6%) did not provide information about the dose of warfarin they took.

No consistent monitoring plan was provided for patients who were given LMWH as an anticoagulation agent.

Data generated from the monitoring plan of patients who received warfarin were provided. There was a variation in the frequency of monitoring the INR levels, ranging from none to weekly. Seventy-five out of the 92 patients (81.5%) taking warfarin were monitored for INR levels during all 3 trimesters of pregnancy, while the rest of the patients (n = 18, 19.5%) were erratically monitored for INR levels during pregnancy. There was a significant difference regarding the monitoring rate of INR levels at all pregnancy stages between the patients undergoing prenatal care in our hospital and the patients referred to our hospitals from elsewhere (86.7% [52/60] vs 68.8% [22/ 32], respectively, *P* = .04). The number of patients who reported INR levels were 56 (56/63, 88.9%) in the 1st trimester, 63 (63/72, 87.5%) in the 2nd trimester, and 60 (60/63, 95.2%) in the 3rd trimester, respectively. The average INR levels were 1.6 ± 0.6 in the 1st trimester, and the median INR levels were 2.0 (1.5–2.0) and 2.0 (1.6–2.0) for the 2nd trimester and the 3rd trimester, respectively.

Before delivery, the following anticoagulation regimens are used among the patients who researched delivery, including planning bridging with LWMH and/or discontinuation LWMH 12 to 24 hour before delivery, planning discontinuation warfarin 3 to 5 days before delivery, continuation of warfarin and continuation of LMWH. For patients with MHVs who reached the 3rd trimester, 49 patients (60.5%) switched from warfarin to LMWH starting from the 36th week of gestation or 3 to 5 days before planned delivery; 16 patients (19.8%) stopped taking LMWH 12 to 24 hours before planned delivery, 7 patients (8.6%) stopped warfarin 3 to 5 days before planned delivery, and 9 patients (11.1%), who experienced urgent CS or spontaneous in-labor, remained original anticoagulant regimen including 4 patients (4.9%) taking warfarin and 5 patients (6.2%) using LMWH when they were in-labor or required urgent operation.

For patients who experienced fetal loss before 28 weeks of gestation, 28 (80%) patients switched from warfarin to LMWH before the planned operation; 1 patients (2.9 %) stopped warfarin directly, and 6 patients (17.1%) stopped LMWH directly.

Warfarin and LMWH were restarted 12 to 24 hours after delivery or termination of pregnancy in all patients with MHVs. When the INR level reached the therapeutic level, LMWH was stopped.

### 3.3. Maternal outcomes

There was 1 maternal death (0.8% of MHVs, 0.7% of PHVs) found in our study. The patient died from heart failure after emergency cardiac surgery for valve thrombosis. The maternal morbidity rate was 26.1% in this study. The main maternal complications included valve thrombosis (10, 7.2%), heart failure (11, 8.0%), endocarditis (2, 1.4%), atrial fibrillation (13, 9.4%), and hemorrhagic events (13, 9.4%). These complications were more likely found in patients with MHVs, whereas the difference was not statistically significant in our study with regard to the rate of maternal morbidity between patients with MHVs and patients with THVs (28.8% vs 15.0%, *P* = .20). In addition, no statistical differences were detected regarding the rate of maternal hospital admission nor the rate of maternal hospital admission for cardiac reasons between these 2 groups (Table 2).

Valve thrombosis occurred in 10 patients (7.5% of PHVs, 8.2% of MHVs) with MHVs, and no valve thrombosis was found in patients with THVs (Table 2). MVT emerged at all stages of pregnancy and on every possible anticoagulation regimen. However, 50% MVTs (n = 5) occurred in the 3rd trimester, and all 5 patients who experienced MVTs in the 3rd trimester took LMWH without a monitoring plan. Only 20% (n = 2) MVTs occurred in patients taking warfarin, and both of them had lower INR levels (Tables 3 and 4). Clinical characteristics, including older age, atrial fibrillation, monitoring coagulation during pregnancy, anticoagulant strategies, and erratic monitoring, were assessed for their influence on the rate of valve thrombosis. The results demonstrated that the rate of valve thrombosis was significantly different among different anticoagulant regimen groups (3.2% [2/63] in warfarin, 0% [0/29] in LMWH/warfarin, 33.3% [8/23] in LMWH, *P*<.01), between the anticoagulation regimens containing warfarin and the regimen of “LMWH throughout pregnancy” (2.2% [2/92] vs 33.3% [8/23], *P*<.01), and between the patients monitoring INR in every trimester of pregnancy and the patients erratically monitoring INR or anti- Xa during pregnancy (1.3% [1/76] vs 22.5% [9/40], *P*<.01). However, when multivariate regression analysis was performed, there were no statistically significant risks of valve thrombosis associated with these factors.

**Table 3:** Outcomes of pregnancy in 3 anticoagulant regimens.

	Warfarin (n = 63), n (%)	LMWH-warfarin (n = 29), n (%)	LMWH (n = 23), n (%)	*P*	Effect size (Cohen’s d)
Age					
Age ≥ 35 yr	16 (25.4)	5 (17.2)	6 (25.0)	.70	0.09
Maternal outcome					
Maternal death	0 (0)	0 (0)	1 (4.3)	.20	0.15
Thrombosed valve	2 (3.2)	0 (0)	8 (33.3)	<.01	0.84
Hemorrhagic events	7 (11.1)	2 (6.9)	3 (12.5)	.75	0.07
postpartum hemorrhage	3 (4.8)	1 (3.4)	0 (0)		
Intra-abdominal bleed	0 (0)	0 (0)	1 (4.2)		
Placenta bleeding	4 (6.3)	1 (3.4)	2 (8.3)		
Fetal outcomes					
Fetal loss	31 (49.2)	2 (6.9)	6 (25.0)	<.01	0.86
Miscarriage < 28 wk	24 (38.1)	1 (3.4)	4 (16.7)	<.01	0.85
Miscarriage < 14 wk	18 (28.6)	0 (0)	3 (12.5)	<.01	0.88
Miscarriage 14–27^+6^ wk	6 (9.5)	1 (3.4)	1 (4.2)		
Stillbirth (≥28 wk)	2 (3.2)	1 (3.4)	0 (0)	1.00	0.06
Therapeutic abortion: maternal condition	2 (3.2)	0 (0)	2 (8.3)	1.00	0.11
Therapeutic abortion<14 wk	2 (3.2)	0 (0)	1 (4.2)		
Therapeutic abortion 14–27^+6^ wk	0 (0)	0 (0)	1 (4.2)		
Therapeutic abortion and termination of pregnancy: fetal condition	3 (4.8)	0 (0)	0 (0)	.42	0.13
Therapeutic abortion 14–27^+6^ wk	2 (3.2)	0 (0)	0 (0)		
Termination of pregnancy ≥ 28 wk	1 (1.6)	0 (0)	0 (0)		
Livebirths	32 (50.8)	27 (93.1)	18 (75.0)	<.01	0.83
Preterm birth < 37 wk	4 (12.5)	3 (11.1)	6 (33.3)	.14	0.21
Median pregnancy duration, wk (Q1–Q3)	38 (37–38)	37 (37–38)	35.5 (35.5–37)	.01	0.75
Median birth weight, g (Q1–Q3)	2955 (2748–3168)	3110 (2720–3280)	2945 (2173–3028)	.16	0.30
SGA	1 (3.1)	1 (3.7)	1 (5.6)	1.00	0.05
Apgar < 7 at 5 min	0 (0)	0 (0)	1 (5.6)	.23	0.18
Neonatal death	1 (3.1)	0 (0)	1 (5.6)	.71	0.09

LMWH = low-molecular-weight heparin, SGA = small for gestational age.

**Table 4:** Mechanical valve thrombosis.

Patient		Age, yr	Valve position	Onset of MVT, gestational wk	AC and dose	INR	Treatment	Pregnancy duration, wk	Delivery	Maternal death	Fetal outcome
1	37		MV	6	Warfarin, 3 mg	1.83	Emergency surgical valve replacement	7	–	NO	Therapeutic abortion
2	25		MV	28	Dalteparin 5000 IU, q12h	–	Emergency surgical valve replacement	28	CS*	Yes, after surgery	Neonatal asphyxia, and neonatal death
3	31		AV	30	Enoxaparin 60 mg, q12h	–	Enoxaprin 60 mg, q8h	37	CS†	NO	Livebirth
4	28		AV	35	Fraxiparine, 0.6 mL, q12h	–	Back to warfarin, 3 mg, qd	37^+^	CS*	NO	Livebirth
5	36		MV	28	Warfarrin, 1.5–2.25 mg, qod	1.37	Surgical valve replacement	28	CS*	NO	Stillbirth 1 d after surgery
6	26		MV	10	Fraxiparine, 0.4 mL, q12h	–	Surgical valve replacement	10	–	NO	Therapeutic abortion
7	35		MV	30	Fraxiparine, 0.6 mL, q12h	–	Warfarin 6 mg, fraxiparine 0.4 mL, q12h	34	CS†	NO	Livebirth
8	29		MV	15	Dalteparin, 6000 IU, q12h	–	Surgical valve replacement at 15 wk	18	–	NO	Therapeutic abortion at 18 wk
9	24		MV	19	Dalteparin, 5000 IU, q12h	–	Surgical valve replacement at 19 wk	38	CS*	NO	Livebirth
10	36		AV	26	Fraxiparine, 0.6 mL, q12h	–	Surgical removal of valve vegetation at 26 wk	36^+6^	CS†	NO	Livebirth

AC = anticoagulation, AV = aortic valve, CS = cesarean section, INR = international normalized ratio, MV = mitral valve, MVT = mechanical valve thrombosis.

* = Cesarean for cardiac reason.

† = Cesarean for obstetric reason.

There was a nondifference in terms of the rate of hemorrhagic events among the 3 anticoagulation regimens (Table 3). Among the 81 patients who reached the 3rd trimester, 5 patients (6.2 %), including 2 patients with LMWH bridging but not stopping LMWH before delivery, 2 patients with warfarin continuation and 1 patient with LMWH, experienced postpartum hemorrhage or intra-abdominal bleeding, whereas no postpartum hemorrhage occurred in patients who stopped LMWH or warfarin 12 to 24 hours or 3 to 5 days before planned delivery, respectively. There was a significant difference in terms of the incidence of postpartum hemorrhage among patients planning stopping anticoagulant and those temporary withdrawal anticoagulant (*P* = .01). Among the 35 patients who experienced miscarriage or termination of pregnancy before 28 weeks of gestation, only 1 patient (2.9%) who had a bridging with LMWH experienced hemorrhage.

### 3.4. Fetal outcomes

Patients with an MHV were significantly less likely to have pregnancies resulting in a live birth compared to those patients with a THV (*P* < .01). Both the rate of miscarriage and the rate of stillbirth were much higher in patients with MHVs than those in patients with THVs (27.1% vs 0%, *P* < .01; Table 2). In the 99 cases of live birth, the median pregnancy duration (*P* = .04) and median birth weight of the baby (*P* = .03) were significantly different between the patients with MHVs and the patients with THVs (Table 2). The chance of having a live birth was 50.8% (n = 32) in patients using “warfarin only” regimen, 93.1% (n = 27) in patients using the “1st-trimester LMWH with subsequent warfarin” regimen, and 75.0% (n = 18) in patients using “LMWH throughout gestation” (*P* < .01) (Table 3). Older age and taking warfarin in the 1st trimester were the risks factors for fetal loss (older age: *P* = .03, OR: 3.083, 95% CI: 1.145–8.032; warfarin in the 1st trimester: *P* < .01, OR: 6.776, 95% CI: 2.662–17.246). With respect to the anticoagulant regimens, “warfarin throughout pregnancy” was the highest risk factor for fetal loss after adjustment by age (warfarin throughout pregnancy vs LMWH: *P* = .04, OR 3.054, 95% CI: 1.040–8.968). The risk of fetal loss in patients with “LMWH in the 1st trimester following warfarin in the 2nd and 3rd trimesters” was similar to that in patients with “LMWH throughout gestation” (*P* = .10, OR 0.234, 95% CI: 0.042–1.319). In regard to the babies born before 37 weeks of gestation, 6 patients (42.9%) were due to spontaneous preterm labor, and the other 8 (57.1%) were elective delivery (5 due to maternal cardiac reasons and 3 for maternal obstetric reasons).

Five fetuses and 1 neonate were diagnosed with congenital abnormalities, with four of them occurring in patients taking warfarin in the 1st trimester and 2 occurring in patients receiving LMWH in the 1st trimester (Table 5).

**Table 5:** Fetal anomalies related to anticoagulation regimen in pregnancy.

Fetus	Ultrasonic diagnosis	Gestational age at diagnosis, wk	Anticoagulant regimen	Dose of warfarin, mg/d
1	Fatal death, abnormalities of intracranial structure (unclear cerebellar structure, absence of bilateral thalamus and septum pellucidum, and absence of gyrus)	22	Warfarin throughout pregnancy	6
2	Fetal hydrocephalus	30	Warfarin throughout pregnancy	4.5
3	Fetal death, fetal hydrocephalus	22	Warfarin throughout pregnancy	4.5/5.25, qod
4	Endocardial cushion defect, hypoplastic right ventricle, permanent left superior vena cava, superabodominal cystic mass,	25	Warfarin throughout pregnancy	3
5	Intraventricular hemorrhage, choroid plexus papillomas	37	First trimester LMWH with subsequent warfarin	4
6	Ventricular septal defect	neonatal period	First trimester LMWH with subsequent warfarin	No record

LMWH = low-molecular-weight heparin.

## 4. Discussion

### 4.1. Main findings

This manuscript presented data on the medical management of pregnant patients with prosthetic valves at a medical center specialized in pregnancy and cardiac diseases. In this single-centered study, 118 pregnant women with prosthetic valves were included; the maternal mortality rate was 0.7%, the fetal loss rate was 28.3%, and valve thrombosis and hemorrhagic complications rates were 0.7% and 9.4 %, respectively. Although the maternal mortality and morbidity of serious complications during pregnancy with PHVs in this study were lower than those in previous reports for this population, adverse fetal outcome frequency remains relatively high, especially in patients with MHVs. Anticoagulation regimens that included warfarin use were associated with a higher fetal loss rate, whereas these warfarin-containing anticoagulation regimens were also associated with a lower MVT rate.

### 4.2. Maternal and fetal outcomes in patients with prosthetic heart valves

The rates of maternal mortality and serious maternal morbidity were low, which were 0.7% and 26.1%, respectively. About 7.2% of the patients in this study experienced valve thrombosis, 11.8% suffered from heart failure, 1.4% had endocarditis, 9.4% suffered from atrial fibrillation, and 9.4% had an obstetric hemorrhage. The rates of these adverse maternal outcomes are lower than those reported in a previous systematic review (maternal mortality: 1.8 death per 100 pregnancies, obstetric hemorrhage:11.1%; and thromboembolic events: 13.9%).^[5]^

In our study, the maternal outcomes in patients with MHVs showed a trend of being worse than that of patients with this, although the difference was not statistically significant in our study. This result is similar to previous reports.^[5,6]^ The rate of MVT in our study is essentially in agreement with the results in previous literature,^[7,8]^ in which it varied from 4.7% to 14.9%.^[5,6,9,10]^ We found a relatively low percentage of hemorrhagic events (10.2%) in our study compared to other studies, in which the rates of hemorrhage varied from 23.1% to 29%.^[6,11]^

In this study, the fetal loss occurred in 28.3% of all the pregnant patients with PHVs, whereas in patients with mechanical prosthesis valves, it was higher (33.1%). The rate of fetal loss dramatically varied from 17% to 63% in previous studies,^[6,12,13]^ which may be attributed to the differences in study population and methods used in these studies.^[6,12,13]^ In our study, a larger percentage of pregnant patients had mechanical valves, which was more likely associated with higher fetal loss in previous literature.^[5,6]^

### 4.3. Effect of anticoagulation on maternal and fetal outcomes

Literature^[5,6,10]^ has consistently demonstrated better maternal outcomes associated with warfarin and better fetal outcomes with LMWH. In fact, there is no single optimal anticoagulation strategy that suits all women.^[3]^ In the literature, heparin, specifically, Unfractionated heparin (UFH), was associated with an increased thrombotic risk.^[5,6,10]^

In our study, most patients used subcutaneous LMWH at a fixed dose without monitoring the anti-Xa levels, and 80% of thrombosis valves occurred in patients using LMWH. The result is partially attributed to the lack of a monitoring plan and the failure to adjust the dose of LMWH during pregnancy. Previous studies also showed that the thrombotic events were associated with the improper application or erratic monitoring of LMWH or poor patient compliance.^[11,13–16]^ In addition, women with MHVs need to be treated with a higher starting dose and dose-adjusted LMWH during pregnancy to maintain an adequate anti-Xa level,^[11,17]^ partially due to the hypercoagulative state^[18]^ and the increased glomerular filtration rate during pregnancy.^[17^ In our study, only 2 MVTs were observed in patients using warfarin. Neither of them received effective anticoagulation monitoring, evidenced by the fact that one had a lower level of INR (<2) and the other did not have meticulous monitoring INR. In our study, maternal outcomes are better in the patients taking warfarin, which is attributed to the effective monitoring methods and the effective maintenance of the coagulation state recommended for MHVs by the guidelines.^[3,19]^

The most extensive prospective observational study on pregnancy outcomes of MHVs found that the risk of miscarriage and late fetal death clearly increased in patients receiving vitamin K antagonist (VKA) in the 1st trimester and in patients receiving VKA throughout gestation.^[6]^ We found a higher rate of fetal loss (49.2%) in patients using warfarin *t* only, most of which occurred before 28 weeks of gestation. Only 1 fetal abnormality occurred in a patient receiving high-dose warfarin in the 1st trimester. Some studies demonstrated that the fetal risk in women taking ≤5 mg daily warfarin was no different from that of the women who LMWH.^[20,21]^ In our study, 85.9% of patients using warfarin received the medicine at a dose of ≤5 mg daily, but the conclusion of fetal risk on warfarin taking was inconsistent with previous studies. This result may be partially due to the relatively small sample size and the larger portion of miscarriages included in our study. Other studies^[6,21,22]^ showed inconsistent results about poor fetal outcomes between women receiving low-dose and high-dose VKA. It is still critical to further explore the effect of warfarin dose on the fetus in the future.

### 4.4. Choice of anticoagulant regimen in pregnant women with MHVs

Choosing an anticoagulation regimen is challenging since there are inherent trade-offs between maternal safety and fetal safety. No clear evidence supports 1 regimen over another among currently available anticoagulation regimens. Our data suggested no anticoagulation regimen was optimally safe for both the mother and the fetus. Based on our data, sequential therapy, which is LMWH during the 1st trimester and warfarin during the 2nd and 3rd trimesters, maybe a relatively better choice for women with MHVs because no MVT occurred in this group of patients, and the fetal loss rate was lower. Although physicians provide careful prepregnancy counseling, the decision can be made only by the mother. Whichever regimen is chosen, it is clear that anticoagulation treatment increases maternal and fetal risks. The effectiveness of anticoagulation should be closely monitored, and the dose of anticoagulants should be adjusted according to the monitoring index.^[6,11]^

### 4.5. Management of pregnant women with PHVs

Compared to pregnant women with tissue valves, those with MHVs might experience more cardiac complications. Pregnancies with MHVs should be managed by a multidisciplinary team in a specialized medical center,^[19]^ which provides prepregnancy assessment and counseling, surveillance during pregnancy, and perinatal management. Planned delivery is recommended for women with MHVs.^[19]^ The delivery plan should involve the timing and the method of delivery, anesthetists, or analgesia. Our data showed that patients with MHVs in a specialized medical center for pregnancy and cardiac disease were monitored for INR more frequently. A high rate of cesarean section was observed in our study, partially due to the increase in obstetric needs and the need of urgent operations. It was also observed in previous studies, which varied from 46.6% to 64.8%.^[6,11,22]^ Spinal anesthesia was wildly performed, whereas general anesthesia was used in urgent CS or cases needing cardiac operation at the same time.

## 5. Limitations

This study is a retrospective, single-center analysis with data extracted from medical records. As some patients were referred to our hospital in the later stages of pregnancy, we often lacked accurate information regarding events that occurred in earlier stages of pregnancy or before pregnancy, such as prepregnancy counseling and the timing of anticoagulant medication changes. Additionally, some data were incomplete or unavailable, such as the frequency of INR monitoring. In this study, patients using LMWH did not receive the same coagulation status monitoring regarding effectiveness as those using warfarin, which could lead to potential bias in the conclusions. Furthermore, differences in monitoring and dose adjustments between LMWH and warfarin during pregnancy may impact the evaluation of anticoagulation effectiveness, further influencing the reliability of the results.

Another significant limitation is the relatively small sample size, which limits the statistical power of the study to detect clinically important differences between groups. The small sample size also restricted the ability of the study to evaluate potential confounding factors, such as underlying cardiac diseases or obstetric complications, which may affect pregnancy outcomes. Although the data suggest that sequential therapy may be associated with better maternal and fetal outcomes compared to other anticoagulation regimens, this finding cannot be definitively proven due to the small sample size and the retrospective design. While the results align with existing literature, they must be interpreted with caution, as the study design does not allow for causal inferences.

Moreover, since the study was conducted at a single-center, the findings may not be widely generalizable to other populations or regions, limiting the external validity of the conclusions. The conclusions drawn from this study may not fully reflect the broader population of women with PHVs and pregnancy.

Given these limitations, future studies should consider adopting prospective research designs and multicenter collaborative studies to increase the sample size, statistical power, and generalizability of the findings. Multicenter studies would help reduce the biases associated with single-center research and ensure that the results are more applicable to diverse patient populations. Additionally, the implementation of standardized anticoagulation monitoring protocols for both LMWH and warfarin, including frequency of monitoring and dose adjustments, would enhance the consistency and reliability of future research findings.

Furthermore, future research should focus on assessing potential confounding factors, such as underlying cardiac conditions, obstetric complications, maternal age, and other demographic characteristics, as these may influence the outcomes of different anticoagulation regimens. By increasing the sample size and conducting a more detailed analysis of these factors, future studies could help confirm the findings of this study and provide more robust evidence for clinical practice.

## 6. Conclusion

Patients with a tissue valve were at fewer risks of fetal mortality and morbidity, while patients with MHVs were at increased risks of fetal loss, particularly miscarriage during the 1st trimester. This study analyzed the effects of 3 anticoagulation regimens on maternal and fetal outcomes. The regimen of “warfarin only” resulted in the highest rate of miscarriage and fetal death, whereas MVTs occurred in the other 2 anticoagulation regimen groups, with 80% of MVTs in women on LMWH. When the outcomes of both mother and fetus are considered, the regimen of “sequential therapy, with LMWH during the 1st trimester and warfarin during the 2nd and 3rd trimesters” may be superior. It is important for women with prosthetic valves to seek prepregnancy counseling with a specialist, and receive adequate guidance and care throughout the gestation, during the delivery, and the postpartum period from a specialized, multidisciplinary medical team.

## Acknowledgments

We would like to thank Professor Tie Zheng, who helped us during the management of these patients.

## Author contributions

**Conceptualization:** Dong Yang, HaoFeng Zhang, Jiaqi Zeng, Xiaojun Liang, Lianmei Luo, Yu Song, Baiyu Tian, Yanna Li, Jie Han, Jun Zhang.

**Data curation:** Dong Yang, HaoFeng Zhang, Jiaqi Zeng, Xiaojun Liang, Lianmei Luo, Yu Song, Baiyu Tian, Yanna Li.

**Formal analysis:** Dong Yang, HaoFeng Zhang, Xiaojun Liang, Jun Zhang.

**Funding acquisition:** Xiaojun Liang, Jun Zhang.

**Investigation:** Dong Yang, HaoFeng Zhang, Jiaqi Zeng, Xiaojun Liang, Lianmei Luo, Yu Song, Baiyu Tian, Yanna Li, Jun Zhang.

**Methodology:** Dong Yang, HaoFeng Zhang, Jiaqi Zeng, Xiaojun Liang, Lianmei Luo, Yu Song, Baiyu Tian, Yanna Li, Jun Zhang.

**Supervision:** Jiaqi Zeng, Jie Han.

**Validation:** HaoFeng Zhang, Xiaojun Liang, Baiyu Tian, Yanna Li, Jie Han, Jun Zhang.

**Visualization:** Dong Yang, HaoFeng Zhang, Xiaojun Liang, Yu Song, Baiyu Tian, Yanna Li, Jie Han, Jun Zhang.

**Writing – original draft:** Dong Yang, Jun Zhang.

**Writing – review & editing:** Dong Yang, Jun Zhang.
